# Evaluation of Susceptibility Testing Methods for Aztreonam and Ceftazidime-Avibactam Combination Therapy on Extensively Drug-Resistant Gram-Negative Organisms

**DOI:** 10.1128/AAC.00846-21

**Published:** 2021-10-18

**Authors:** Ayesha Khan, Samuel G. Erickson, Cedric Pettaway, Cesar A. Arias, William R. Miller, Micah M. Bhatti

**Affiliations:** a Center for Antimicrobial Resistance and Microbial Genomics, Division of Infectious Diseases, UTHealth, McGovern Medical School, UTHealth, Houston, Texas, USA; b Center for Infectious Diseases, UTHealth School of Public Health, Houston, Texas, USA; c Department of Pathology and Laboratory Medicine, The University of Texas MD Anderson Cancer Center, Houston, Texas, USA

**Keywords:** *Enterobacteriaceae*, antibiotic resistance, antimicrobial combinations, beta-lactamases, carbapenems, clinical methods, diagnostics, metalloenzymes, multidrug resistance, susceptibility testing

## Abstract

Carbapenem-resistant *Enterobacterales* (CRE) and Pseudomonas aeruginosa (CR-PA) producing metallo-β-lactamases (MBLs) cause severe nosocomial infections with no defined treatment. The combination of aztreonam (ATM) with ceftazidime-avibactam (CZA) is a potential therapeutic option, but there is no approved, feasible testing method for use in clinical laboratories to assess the activity of two antimicrobials in combination. Here, we evaluate the performance of four ATM-CZA combination testing methods, as follows: broth disk elution (DE), disk stacking (DS), strip stacking (SS), and strip crossing (SX). We used 10 clinical, representative *Enterobacterales* and 6 P. aeruginosa isolates harboring MBL, Guiana extended-spectrum beta-lactamase (GES), or non-MBL enzymes. Four of these isolates were from clinical cases treated by ATM-CZA. All CRE producing NDM and CR-PA producing GES that were resistant to ATM and CZA alone were susceptible to the ATM-CZA combination. P. aeruginosa generating NDM or VIM remained resistant to ATM-CZA, likely due to non-β-lactamase mechanisms, and all other isolates were susceptible to ATM or CZA alone. The most accurate, precise, and reproducible methods of low complexity were disc elution and both strip methods (SX and SS) using MIC test strips (MTS) , all with 100% sensitivity and specificity, followed by Etest with SX (95.83% sensitivity, 100% specificity) and SS (87.5% sensitivity, 100% specificity). DS had the lowest performance. DE is particularly valuable in low-resource settings that routinely use disks. MTS yielded higher categorical agreements by SX (94%) and SS (84%), relative to Etest by SX (90%) and SS (82%). P. aeruginosa results yielded the majority of the errors. These methods may allow laboratories to inform clinical decision making like combination therapy for severe infections caused by extensively drug-resistant *Enterobacterales.*

## INTRODUCTION

Antimicrobial resistance (AMR) is recognized as a major global health threat by the World Health Organization and the Centers for Disease Control and Prevention, causing over 35,000 annual deaths in the United States alone (1, 2). Carbapenem-resistant *Enterobacterales* (CRE) and Pseudomonas aeruginosa (CR-PA) have become serious causes of recalcitrant health care-associated infections that are often resistant to common therapeutic options (3). Bloodstream infections due to CRE and CR-PA are associated with a >30% mortality rate (4). Rapid global dissemination of CRE and CR-PA is often due to organisms acquiring and sharing AMR determinants. Organisms that are carbapenem resistant due to the production of metallo-beta-lactamases (MBLs), like the New Delhi MBL (NDM), Verona integron-encoded MBL (VIM), and imipenem-hydrolyzing MBL (IMP) are particularly problematic. These enzymes can inactivate the majority of beta-lactams in clinical use, except for monobactams like aztreonam (ATM). Cefiderocol, one of the latest antimicrobials introduced to clinical settings, also has decreased activity in strains producing NDM compared with other MBL enzymes (5). Carbapenem resistance in P. aeruginosa is most commonly mediated by noncarbapenemase mechanisms that include the overproduction of AmpC, porin loss, and drug efflux, although some isolates also produce carbapenemases (6). Indeed, P. aeruginosa harboring MBLs and variants of the Guiana extended-spectrum beta-lactamases (GESs), which have a hydrolysis profile similar to MBLs, are an emerging threat (7, 8).

There is no defined optimal treatment regimen for MBL-producing organisms. While agents like colistin and polymyxin B are utilized based on *in vitro* susceptibility, their clinical utility is questionable due to a narrow therapeutic window leading to toxicity and emergence of resistance during therapy (9). Aztreonam is stable in the presence of MBL enzymes, but its frequent cocarriage of extended-spectrum β-lactamases limits its utility as a monotherapy. Case reports have shown that addition of the novel β-lactamase inhibitor avibactam, via administration of ceftazidime-avibactam (CZA), in combination with ATM (ATM-CZA), may have clinical utility in treating infections caused by MBL-producing CRE and GES-producing CR-PA (7, 10–12). These findings were bolstered by a recent prospective observational study of patients with bloodstream infections due to MBL-producing *Enterobacterales* that showed that ATM-CZA may have a therapeutic advantage, with a 60% reduction in risk of mortality compared with other active antibiotics (hazard ratio, 0.37 [95% confidence interval, 0.13 to 0.74]; *P *= 0.01), lower clinical failure at day 14, and a shorter length of hospital stay (13). Avibactam is a novel, potent inhibitor of class A and C beta-lactamase enzymes paired with ceftazidime. ATM, the monobactam, is stable in the presence of MBL enzymes. Thus, the combination is likely efficacious because avibactam inhibits class A and C enzymes from hydrolyzing ATM, and ATM is intrinsically able to evade class B enzymes and retain antimicrobial killing activity.

Currently, there is no practical and widely accepted antimicrobial susceptibility testing (AST) method available for use in clinical laboratories for assessing the efficacy of the ATM-CZA combination. Established testing methods to evaluate combinations like the checkerboard assay or time-kill assays are complex, labor intensive, and difficult to interpret. Simpler methods like the crossing of gradient strips are highly subjective, prone to human error, and do not correlate well with better-established methods (7, 10–12). Clinical case studies demonstrating the utility of the CZA and ATM combination against MBL-producing *Enterobacterales* and Stenotrophomonas maltophilia or GES-producing P. aeruginosa used various susceptibility testing methods to inform treatment decisions. These studies included the use of disk stacking (DS), gradient strip stacking (SS), and ATM gradient strips placed on Mueller-Hinton agar supplemented with CZA to assess the efficacy of the ATM-CZA combination. A newer method for MIC determination, designated disk elution (DE), combines the ease of disk diffusion with the reliability of broth dilution methods. Disk elution was recently endorsed by CLSI as an acceptable method for determining the susceptibility of Gram-negative bacilli to colistin (14, 15). To date, there are no published studies using the disk elution methodology for combination testing.

In this study, the *in vitro* performance of ATM-CZA susceptibility testing was assessed using two disk-based methods, namely, disk stacking (DS) and broth disk elution (DE), and two MIC-based methods, namely, gradient strip stacking (SS) and gradient strip crossing (SX). The rationale for evaluating these procedures was to find an accurate method of low complexity that can be routinely implemented in clinical laboratories, as well as one that is affordable for use in resource-limited settings.

## RESULTS

### Establishing a gold standard and characterization of strains.

In order to evaluate the performance of four combination testing methods, a cohort of representative strains were selected with predictable responses to the ATM-CZA combination based on their genotypes. The cohort included eight *Enterobacterales* and eight P. aeruginosa strains harboring MBL (NDM, VIM, and IMP) and non-MBL enzymes (GES, OXA-48, and KPC) (Table 1). Three of these isolates, namely, Escherichia coli, Klebsiella pneumoniae harboring NDM-5, and a P. aeruginosa harboring GES-19 and GES-26, were isolated from patients treated successfully with ATM-CZA combination therapy and characterized in previous studies (7, 10). A modified broth microdilution (mBMD) method was selected as our reference method for MIC determination, as this technique is preferred for evaluating the performance of antimicrobial susceptibility testing and is endorsed by laboratory guidelines (16). The MIC of ATM was determined in the presence or absence of a constant concentration of CZA (4 μg/ml of the avibactam component), which was chosen to be consistent with the concentration of avibactam in gradient test strips. This concentration is also close to the serum nadir of avibactam (2.2 μg/ml) from human pharmacokinetic data (17–19). All mBMD testing for each strain was performed in triplicate on different days. The interpretation of the ATM MICs (i.e., resistant, intermediate, and susceptible) was based on current CLSI M100 breakpoints (20) (Table 2). If an isolate, resistant to both ATM alone and CZA alone, yielded a susceptible ATM MIC in the presence of CZA, it was categorized as “synergy positive” (Table 1). If an isolate was nonsusceptible to both ATM alone and CZA alone and the ATM MIC remained in the resistant range in the presence CZA, it was categorized as “synergy negative” (Table 1). If an isolate was susceptible to ATM alone and/or CZA alone, it was automatically categorized as synergy negative. Additionally, we calculated the fractional inhibitory concentration (FIC) for ATM in the presence of CZA (Table 3). An FIC of ≤0.5 correlated with the isolates which were synergy positive, while an FIC of >0.5 correlated with a lack of synergy (14). The mBMD testing, supported by the FIC, determined the susceptibility of each isolate to the ATM-CZA combination and established a reference for use in assessing the performance of the test methods.

**TABLE 1:** Characteristics of strains used in this study^*a*^

Strain name (reference)	β-Lactamase(s)	Agent	mBMD MIC (μg/ml)	S/I/R^*b*^	Synergy category	CDC MIC (μg/ml)
Escherichia coli ATCC 25922	None, quality-control strain	ATM	<0.25	S	Negative	
		CZA	<0.25	S		
		ATM + CZA	<0.25	S		
E. coli 2769 (10)	NDM-5	ATM	>64	R	Positive	
		CZA	>64	R		
		ATM + CZA	2	S		
Klebsiella pneumoniae 2770 (10)	NDM-5	ATM	>64	R	Positive	
		CZA	>64	R		
		ATM + CZA	0.5	S		
Pseudomonas aeruginosa HTX_1 (7)	GES-19 and GES-26	ATM	>64	R	Positive	
		CZA	>64	R		
		ATM + CZA	4	S		
P. aeruginosa **HTX_70**	VIM-2	ATM	16	I	Negative	
		CZA	>64	R		
		ATM + CZA	16	I		
P. aeruginosa HTX_133	GES-19 and GES-26	ATM	>64	R	Positive	
		CZA	32	R		
		ATM + CZA	4	S		
K. pneumoniae **622**	VIM	ATM	<0.25	S	Negative	
		CZA	32	R		
		ATM + CZA	<0.25	S		
Enterobacter cloacae 1042 (CDC AR bank mero/vab panel)	NDM	ATM	>64	R	Positive	>64
		CZA	>64	R		>16
		ATM + CZA	1	S		
P. aeruginosa **0241** (CDC AR bank PA panel)	IMP-1	ATM	8	S	Negative	32
		CZA	>64	R		>16
		ATM + CZA	8	S		
E. coli 1055 (CDC AR bank mero/vab panel)	NDM	ATM	>64	R	Positive	>64
		CZA	>64	R		>16
		ATM + CZA	8	S		
E. coli 1057 (CDC AR bank mero/vab panel)	NDM	ATM	>64	R	Positive	32
		CZA	>64	R		>16
		ATM + CZA	4	S		
K. pneumoniae 1063 (CDC AR bank mero/vab panel)	NDM, OXA-48	ATM	>64	R	Positive	>64
		CZA	>64	R		>16
		ATM + CZA	0.5	S		
P. aeruginosa **0246** (CDC AR bank PA panel)	NDM-1	ATM	>64	R	Negative	>64
		CZA	>64	R		>16
		ATM + CZA	64	R		
P. aeruginosa **0250** (CDC AR bank PA panel)	NDM-1	ATM	>64	R	Negative	>64
		CZA	>64	R		>16
		ATM + CZA	>64	R		
P. aeruginosa **0239** (CDC AR bank PA panel)	VIM-11	ATM	8	S	Negative	32
		CZA	>64	R		>16
		ATM + CZA	4	S		
P. aeruginosa **0249** (CDC AR bank PA panel)	VIM-2	ATM	16	I	Negative	32
		CZA	>64	R		>16
		ATM + CZA	16	I		
K. pneumoniae **1041** (CDC AR bank mero/vab panel)	OXA-48	ATM	64	R	Negative	64
		CZA	2	S		1
		ATM + CZA	1	S		

a Susceptibility to the ATM and CZA combination was assessed by modified broth microdilution (mBMD) using CLSI M100 breakpoint MICs to determine if susceptibility to ATM is restored in the presence of a stable concentration of CZA (4 μg/ml of the avibactam component). Mode of the mBMD biological triplicates is indicated. MICs established by the CDC are reported for isolates obtained from the CDC antimicrobial resistance (AR) meropenem-vaborbactam (mero/vab) or PA panel. ATM resistant (ATM-R) and CZA-resistant (CZA-R) strains where susceptibility to ATM is restored in the presence of CZA are called “synergy positive” and are underlined. Strains in bold are synergy negative and are either ATM-R and CZA-R strains that remained resistant to the combination or strains that are susceptible to ATM or CZA alone.

b S, susceptible; I, intermediate; R, resistant.

**TABLE 2:** Breakpoints used in this study from CLSI M100

Organism	Antimicrobial	MIC breakpoints (μg/ml)			Disk breakpoints^*a*^ (mm)		
		S	I^*b*^	R	S	I^*b*^	R
*Enterobacterales*	ATM	≤4	8^	≥16	≥21	18–20^	≤17
	CZA	≤8		≥16	≥21		≤20
P. aeruginosa	ATM	≤8	16^	≥32	≥22	16–21	≤15
	CZA	≤8		≥16	≥21		≤20

a ATM and CZA CLSI disk breakpoints are based on disks with 30 μg and 20–30 μg of antibiotics, respectively.

b Intermediate ranges denoted with a ^ for aztreonam are based on the known ability of the agent to concentrate in the urine and may also have the potential to concentrate at other anatomical sites.

**TABLE 3:** Average FIC calculated with individual FIC values from the mBMD, SX, and SS triplicates

Strain	Results by assay									
	mBMD		Strip crossing				Strip stacking			
	Synergy^*a*^	FIC	Etest synergy^*a*^	Etest FIC	MTS synergy^*a*^	MTS FIC	Etest synergy^*a*^	Etest FIC	MTS synergy^*a*^	MTS FIC
E. coli 2769	Y	0.026	Y	0.010	Y	0.009	Y	0.006	Y	0.005
K. pneumoniae 2770	Y	0.021	Y	0.007	Y	0.014	Y	0.001	Y	0.002
P. aeruginosa HTX_1	Y	0.188	Y	0.052	Y	0.057	Y	0.052	Y	0.052
P. aeruginosa **HTX_70**	N	1.25	N	0.806	N	0.708	N	0.847	N	1.151
P. aeruginosa HTX_133	Y	0.292	Y	0.063	Y	0.057	Y	0.063	Y	0.042
K. pneumoniae **622**	N	1.010	N	0.902	N	0.091	N	0.902	N	1.003
Enterobacter cloacae 1042	Y	0.026	Y	0.010	Y	0.010	Y	0.007	Y	0.005
P. aeruginosa **0241**	N	1.125	N	0.814	N	1.031	N	0.814	N	1.547
E. coli 1055	Y	0.25	Y	0.073	Y	0.052	Y	0.135	Y	0.052
E. coli 1057	Y	0.104	Y	0.029	Y	0.150	Y	0.023	Y	0.124
K. pneumoniae 1063	Y	0.021	Y	0.010	Y	0.013	Y	0.007	Y	0.004
P. aeruginosa **0246**	N	2	N	2	N	2	N	2	N	2
P. aeruginosa **0250**	N	2	N	2	N	2	N	2	N	2
P. aeruginosa **0239**	N	0.75	N	0.722	N	0.531	N	0.75	N	1.240
P. aeruginosa **0249**	N	1.042	N	0.658	N	0.708	N	0.904	N	1.073
K. pneumoniae **1041**	N	0.516	N	1.035	N	1.375	N	0.648	Y	0.458

a An FIC average of ≤0.5 indicates synergy (Y) between ATM and CZA, while an FIC average of >0.5 indicates the absence of synergy (N) between ATM and CZA.

All six CRE harboring NDM and two CR-PA with GES variants were resistant to ATM and CZA alone but susceptible or intermediate to the ATM-CZA combination (Table 1; see Table S1 in the supplemental material). The average FIC of the strains was ≤0.5 with a range of 0.001 to 0.292, which confirmed the efficacy of the ATM-CZA combination (Table 3). Thus, these isolates were considered synergy positive, with 7 isolates categorized susceptible and 1 E. coli strain (number 1055) as intermediate to ATM in the presence of CZA (Table 1). The remaining 8 isolates were considered synergy negative (Table 1). Four CR-PA strains, namely, two with NDM-1 (0246 and 0250) and two with VIM-2 (0249 and HTX_70), were nonsusceptible to ATM and CZA alone, and the ATM-CZA combination had no effect on the ATM MIC (Table 1; Table S1). The average FIC values of these strains ranged between 0.708 and 2, which confirmed the lack of efficacy of ATM in combination with CZA (Table 3). Three isolates were susceptible or intermediate to ATM alone, and the MIC to ATM did not change in the presence of CZA. One isolate (KP 1041) was resistant to ATM alone (MIC, 64 μg/ml), and the MIC significantly decreased in the presence of CZA to an MIC of 1 μg/ml, but the isolate was also susceptible to CZA alone (MIC, 2 μg/ml). Thus, this isolate was still categorized as synergy negative with a FIC of 0.516.

### Performance of the gradient strip methods.

**FIG 1:**
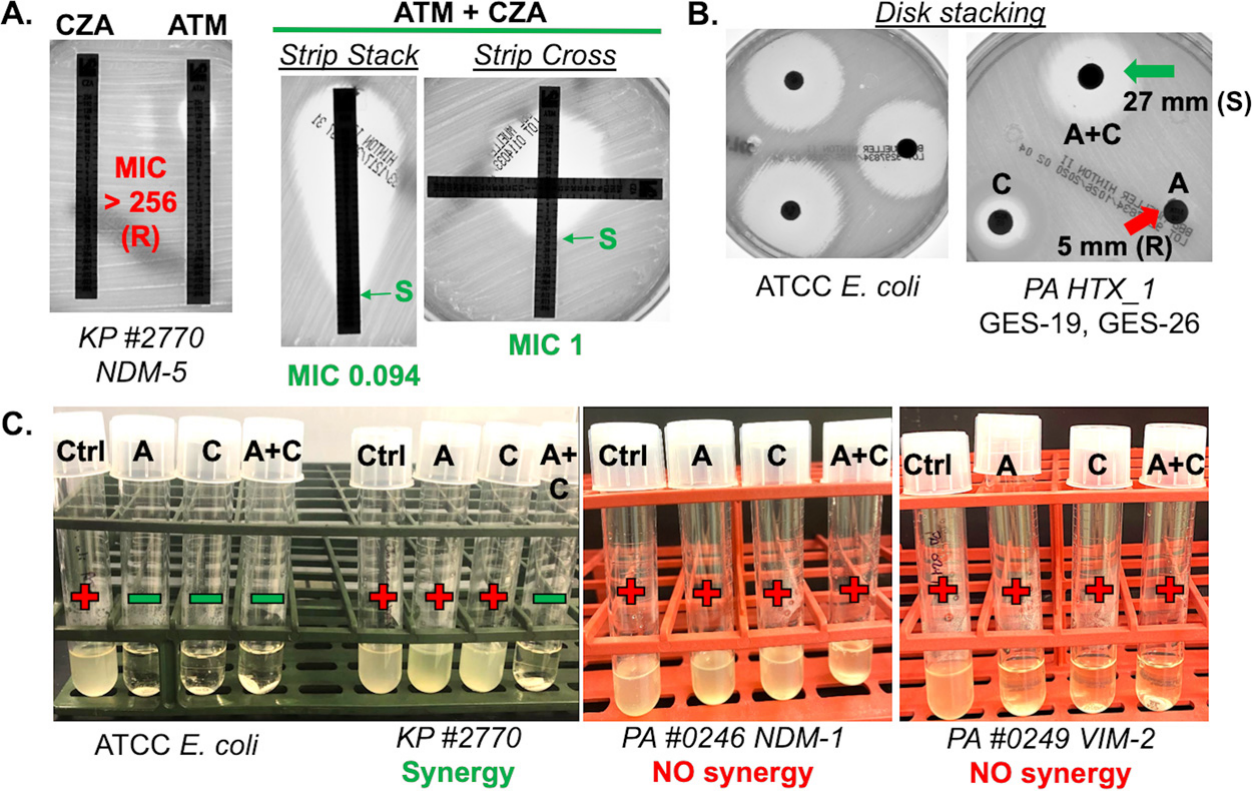
Representative images of combination testing methods. All methods were compared with a modified broth microdilution reference that defines whether or not the ATM-CZA combination is synergistic against a strain. (A) Strip stacking and strip cross methods demonstrated with K. pneumoniae 2770, harboring NDM-5, and resistant to ATM and CZA alone (left; R, red). ATM susceptibility (right; S, green) was restored with the CZA strip present indicating that the ATM-CZA combination is synergistic against the strain via both methods. (B) Disk stacking method demonstrated with P. aeruginosa HTX_1, harboring GES-19, GES-26, resistant to ATM (A) and CZA (C) alone (red arrow). An ATCC E. coli strain was used as a negative control. Efficacy of the ATM-CZA combination (A+C, green arrow) is indicated by the zone diameter categorizing the strain as susceptible to ATM only if the CZA disk is stacked on top. (C) Disk elution method shows that K. pneumoniae 2770 has turbid growth (+) in the tubes with an ATM (A) or CZA (C) disk suspended in 2 ml of Mueller-Hinton broth. Efficacy of the ATM-CZA combination against 2770 was observed by a clearing or absence of any growth (−) in the tube with both an ATM and CZA disk (A+C) suspended in broth. ATM-CZA shows no synergistic activity against two example P. aeruginosa strains indicated by the high density growth (0246) or more faint, low density growth (0249) observed under all three conditions (A, C, and A+C). An ATCC E. coli strain was used as a negative control. An assessment of growth or no growth was made after incubation by visually comparing turbidity to a tube with sterile broth alone.

The gradient strip stacking (SS) and gradient strip crossing (SX) methods assessed the MIC to ATM alone, CZA alone, and ATM in the presence of CZA, in triplicate, using two different commercially available test strips (MIC test strips [MTS], Liofilchem, Inc., Waltham, MA; and Etest, bioMérieux, Inc., Durham, NC) (Fig. 1). The performance of these methods was evaluated qualitatively by determining the categorical agreement (CA) (i.e., synergy positive or synergy negative) between these methods and the reference mBMD method. Each strain’s average FIC with each method was also calculated for comparison (Table 3). The MTS had complete categorical agreement by both SX and SS methods with a sensitivity and specificity of 100% (Table 4). The Etest also had a high categorical agreement by SX with a 95.83% sensitivity and 100% specificity (Table 4). There was one false negative from a replicate of an isolate (EC 1055), resistant to both ATM and CZA, that yielded a lower but still resistant ATM MIC of 16 μg/ml in the presence of CZA (Table S1). Despite the strain having an FIC of 0.073, a clinical laboratory’s reliance on standard breakpoint interpretations led us to categorize this replicate as synergy negative (Table 3).The other two replicates yielded a susceptible (4 μg/ml) and an intermediate (8 μg/ml) ATM MIC in the presence of CZA which were categorized as synergy positive. Etest by SS had a sensitivity of 87.5% and a specificity of 100% (Table 4). The lower sensitivity was due to one isolate (EC 1055) that yielded three false negatives (Table S1). All three replicates yielded an ATM MIC that remained resistant in the presence of CZA according to the CLSI clinical breakpoint for ATM, despite the strain’s FIC of 0.135 (Table S1 and S3 in the supplemental material).

**TABLE 4:** Evaluation of overall qualitative and quantitative performance of combination testing methods compared to mBMD^*a*^

Parameter	Results by assay					
	Disk elution	Disk stacking	Strip stacking		Strip crossing	
			E-test	MTS	E-test	MTS
Sensitivity	100	42.67	87.5	100	95.83	100
Specificity	100	100	100	100	100	100
EA			38/45 (84)	38/45 (84)	42/45 (93)	42/45 (93)
CA	51/51 (100)	22/51 (43)	42/51 (82)	43/51 (84)	46/51 (90)	48/51 (94)
VME		0/7	0/7	0/7	0/7	0/7
ME		16/37 (43)	2/37 (5)	1/37 (3)	2/37 (5)	0/37
MI		13/51 (25)	7/51 (14)	7/51 (14)	3/51 (6)	3/51 (6)

a Sensitivity and specificity were calculated with a 95% confidence interval (CI); values are %. All other values are *n* (%): EA, evaluable essential agreement; CA, categorical agreement; VME, very major error; ME, major error; MI, minor error.

**FIG 2:**
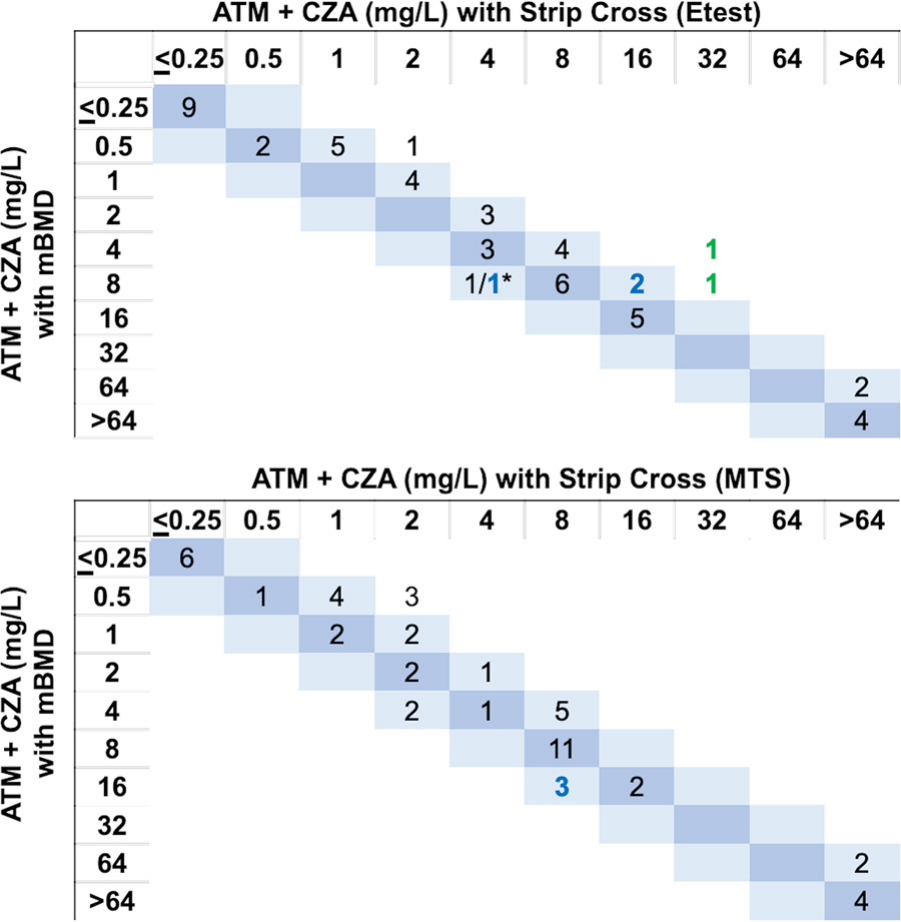
Modified broth microdilution (mBMD) and strip cross (Etest and MTS) distributions of MIC of ATM in the presence of CZA, measured to assess the efficacy of the ATM-CZA combination. Errors based on categorical disagreement are indicated by colors (minor error, blue; major error, green; and very major, red). *, number of P. aeruginosa isolates/number of *Enterobacterales* isolates listed in that order, separated by a back slash; this is to distinguish isolates with errors since ATM breakpoints for are different for Pseudomonas sp. and *Enterobacterales* spp.

**FIG 3:**
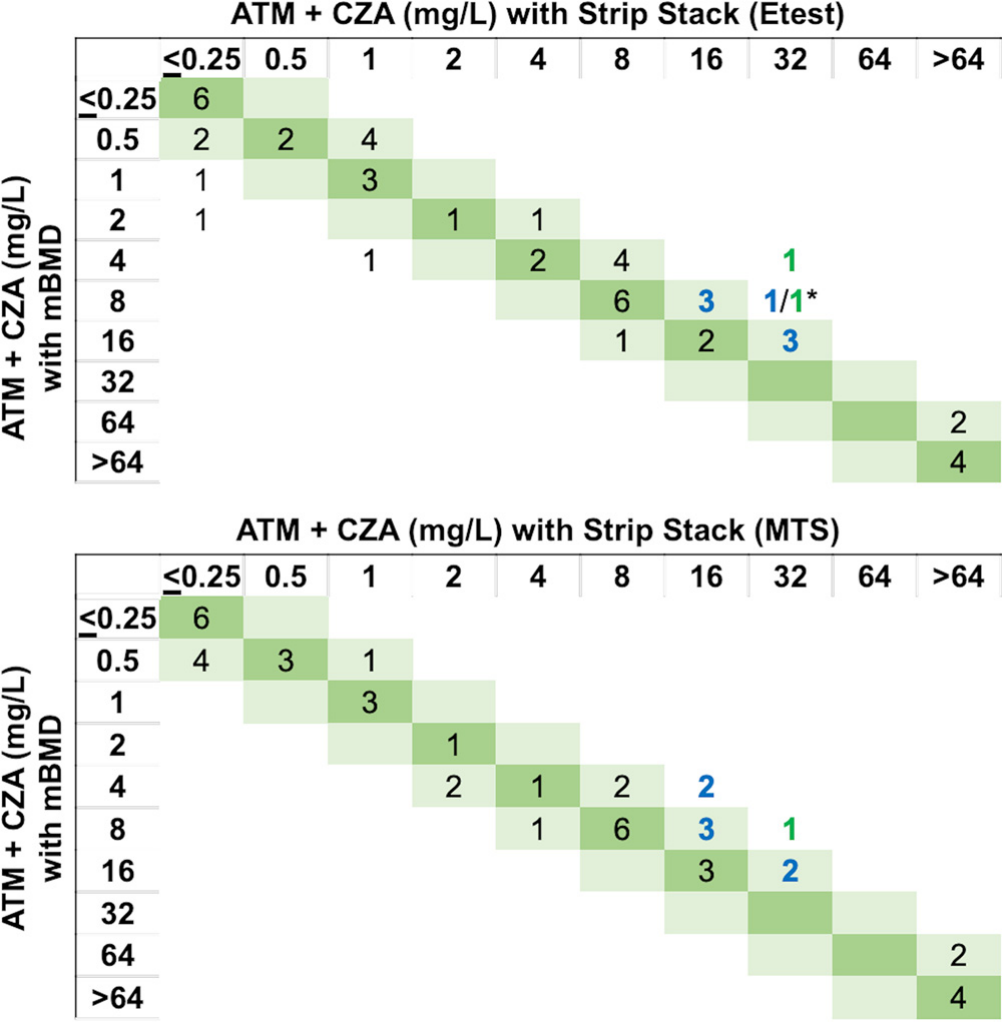
Modified broth microdilution (mBMD) and strip stacking (Etest and MTS) distributions of MIC of ATM in the presence of CZA, measured to assess the efficacy of the ATM-CZA combination. Errors based on categorical disagreement are indicated by colors (minor error, blue; major error, green; very major error, red). *, number of P. aeruginosa isolates/number of *Enterobacterales* isolates listed in that order, separated by a back slash; this is to distinguish isolates with errors since ATM breakpoints for are different for Pseudomonas sp. and *Enterobacterales* spp.

To assess differences in MIC values between SX or SS and mBMD more closely, we performed a quantitative assessment. The SX method by MTS and Etest both had a 93% essential agreement (EA) and a categorical agreement (CA) of 94% and 90%, respectively (Fig. 2, Table 4). The SS method by MTS and Etest both had a 84% EA and a CA of 82% and 84%, respectively (Fig. 3, Table 4). SX by Etest yielded MICs of the ATM-CZA combination (ATM MIC in the presence of CZA) that were 1 log_2_ dilution higher than mBMD MICs for 39% (20/51) of isolates, 1 log_2_ dilution lower for 4% (2/51) of isolates, and 2 log_2_ dilutions or higher (i.e., out of EA) for 6% (3/51) of isolates (Fig. 2). SX by MTS yielded ATM-CZA MICs that were 1 log_2_ dilution higher than the mBMD MICs for 27% (14/51) of isolates, 1 log_2_ dilution lower for 10% (5/51) of isolates, and 2 log_2_ dilutions higher (i.e., out of EA) for 6% (3/51) of isolates (Fig. 2). The SX by Etest yielded 0 very major errors (VMEs), 2 major errors (MEs), and 3 minor errors (MIs), while MTS had 0 VMEs or MEs and 3 MIs (Table 4).

Strip stacking by Etest produced ATM-CZA MICs that were 1 log_2_ dilution higher than the mBMD for 33% (17/51) of isolates, 1 log_2_ dilution lower for 6% (3/51) of isolates, and ≥2 log_2_ dilutions higher or lower (i.e., out of EA) for 12% (6/51) of isolates (Fig. 3). SS by MTS generated ATM-CZA MICs that were 1 log_2_ dilution higher than the mBMD MICs for 20% (10/51) of isolates, 1 log_2_ dilution lower for 14% (7/51) of isolates, and 2 log_2_ dilutions higher for 6% (3/51) of isolates (Fig. 3).The SS by Etest had a 82% CA with 0 VMEs, 2 MEs, and 7 MIs; and by MTS had a 84% CA, 0 VMEs, 1 MEs, and 7 MIs (Table 4).

### Performance of the disk-based methods.

To optimize the DE method, we first determined the approximate concentration of antibiotic that can be eluted out of one Kirby Bauer disk, containing 30 μg of ATM or 20 to 30 μg of CZA, by incubating it in 2 ml of cation-adjusted Mueller-Hinton broth (MHB) for 30 min. This incubation time was chosen based on the CLSI multicenter evaluation study of colistin DE (21). A broth microdilution assay was performed with the quality-control (QC) E. coli ATCC strain that has CLSI-established MIC ranges for ATM and CZA (20). Serial dilutions of the eluted disk broth were used for the mBMD assay. The MIC of ATM and CZA were within the expected range for the ATCC strain (0.125 μg/ml and 0.25 μg/ml, respectively). Thus, the antimicrobial activity in the MHB for DE was roughly equivalent to prepared powder stocks for mBMD. We estimated that the elution of one disk in 2 ml of MHB yields approximately 15 μg of ATM or 15/10 μg of CZA, respectively. This value rounded up yields an ATM concentration at the resistant breakpoint for *Enterobacterales* and intermediate for P. aeruginosa (16 μg/ml). The DE setup involved the following five conditions with 2 ml of MHB in borosilicate tubes: sterile broth, inoculated control, 1 ATM disk, 1 CZA disk, and a combination of 1 ATM plus 1 CZA disk. The inoculum, at a final concentration of 1.5 × 10^5^ CFU/ml of bacteria, was added after the 30-minute elution of the antimicrobial disks, with subsequent incubation at 37°C for 16 to 20 hours, followed by visual inspection for turbid growth (Fig. 1).

The DE method had excellent performance with a 100% sensitivity and specificity (Table 4; Table S1). To verify if one disk was optimal, we performed DE with two disks of ATM and CZA each (final concentration of 30 μg ATM or 20 to 30 μg CZA), in triplicate over 3 days. We used the 5 synergy-positive and 5 synergy-negative isolates (see Table S2 in the supplemental material). The results were in 100% agreement between the DE assay performed with 1 or 2 ATM and CZA disk(s) each.

The DS method had the lowest performance relative to all disk and gradient strip-based methods with 42.67% sensitivity and 100% specificity (Table 4). The 14 false negatives were from the disks incorrectly classifying synergy positive isolates, namely, 1 P. aeruginosa and 5 *Enterobacterales* strains, as resistant to the ATM-CZA combination. By quantitative analysis, DS had a 43% CA, 0 VMEs, 16 MEs, and 13 MIs (Table 4; see Fig. S1 in the supplemental material).

### Reproducibility analysis.

A reproducibility study was performed for the reference mBMD and the highest performing combination testing methods, namely, DE, SX, and SS, over 3 days of testing (Tables 5; Table S1). The mBMD MIC readings for susceptibility to ATM, CZA, and ATM in the presence of CZA all had an EA of 98% to 100%. The DE and SX with MTS had the highest precision of all the methods, with a 100% EA of the ATM-CZA MICs between testing days (Table 5). Overall, all the readings from DE, SX, and SS had an EA between 94% and 100%.

**TABLE 5:** Reproducibility study performed to assess variability of mBMD, DE, SX, and SS methods^*a*^

Method	Total agreement (*n* = 51)			*Enterobacterales* agreement (*n* = 27)			P. aeruginosa agreement (*n* = 24)		
	ATM	CZA	A + C	ATM	CZA	A + C	ATM	CZA	A + C
mBMD	98 (50)	100	98 (50)	100	100	98 (50)	96 (23)	100	100
Disk elution	96 (49)	94 (48)	100	96 (26)	89 (24)	100	96 (23)	100	100
Strip stacking									
Etest	98 (50)	98 (50)	96 (49)	100	100	96 (26)	96 (23)	96 (23)	96 (23)
MTS	98 (50)	100	94 (48)	100	100	93 (25/27)	96 (23)	100	96 (23)
Strip crossing									
Etest			94 (48)			93 (25/27)			96 (23)
MTS			100			100			100

a Isolates were categorized as synergy positive or negative using the mBMD as a reference method. Note that all other combination testing methods required only the reading of susceptibility to the ATM and CZA combination (A+C). All values are % (*n*).

## DISCUSSION

This is the first study assessing five different methods to determine the *in vitro* efficacy of the aztreonam and ceftazidime-avibactam combination. In the past, it has been difficult to demonstrate that two or more methods testing susceptibility to a combination of antimicrobials can yield comparable and reproducible results (14). However, our results indicate that combination testing is feasible by utilizing methods accessible to most clinical laboratories, including those in resource-limited settings. We effectively established mBMD as a reference method for this study (Table 1; Table S1). As expected, it indicated that all of the MBL-producing CRE and the GES-producing CR-PA included in our study exhibited a synergy-positive phenotype (Table 1). The remainder of the CRE *Enterobacterales* isolates were susceptible to ATM or CZA alone. The MBL-harboring CR-PA strains were likely resistant to the ATM-CZA combination due to non-carbapenemase-mediated mechanisms of resistance. Isolates categorized as synergy positive by mBMD had corresponding FIC scores of ≤0.5, while those categorized as synergy negative had FIC scores of >0.5 (Table 3). Despite the accuracy, precision, and reproducibility of mBMD, the method is too laborious for clinical laboratories.

Of the four test methods assessed, DE and SX with MTS were the two most accurate and precise methods for performing ATM-CZA combination testing (Table 4). This study demonstrated successfully the promising potential of DE for antimicrobial combination testing. The DS method had the worst performance of all methods tested due to the disks overcalling nonsusceptibility to ATM-CZA for isolates that were characterized as synergy positive by mBMD (Table 4). Disk diffusion is commonly reported to overcall resistance to other antimicrobial agents as well due to the variable diffusion of the agents through the agar and human errors with measuring zone diameters. SX had a better performance than SS, and the FIC scores calculated with the MIC values generated from both SX and SS matched well with the FIC scores based on the mBMD reference (Table 3). The SX likely performs better due to the complexity of the SS method. SS involves placing the ATM strip on the inoculated MH plate for 10 minutes, removing it, placing the CZA in the same location, and followed by laying the ATM strip on top of the CZA strip. SX is a comparatively simpler process of laying the ATM strip down followed by the CZA perpendicular to the ATM strip at the intermediate breakpoint (Fig. 1). Our study also demonstrated that the MTS gradient strips (Liofilchem) had higher concordance with the reference mBMD than Etest strips (Table 4; Table S1). One possible explanation for the difference in performance is the design of the strips. The MTSs are made from paper, whereas Etest is a plastic-based strip. While either design works appropriately when used as an individual test, the paper of the MTS strips may allow for more efficient diffusion when laid across another strip as opposed to the plastic Etest strips (see Fig. S2 in the supplemental material). This explanation is supported by the observation of individual colonies within the inner zone of clearance on several of the SX and SS agar plates using Etest strips, while the MTS plates consistently had a clear inner zone as shown in Fig. S2. While this result is intriguing for clinical labs in general, additional testing with a larger sample size is needed to confirm this finding.

The primary goal of the study was to assess methods for testing the efficacy of two agents in combination that would be accessible to clinical microbiology laboratories. While adding to the susceptibility testing toolbox is important, it is equally important to know when these tools will be the most useful. As part of the ATM-CZA combination, ATM is not susceptible to hydrolysis by MBLs and is protected from ESBLs by avibactam. There is a high global prevalence of CRE coharboring MBLs and ESBLs on mobile genetic elements (22–24). The ATM-CZA combination was shown to have clinical utility for the treatment of bacteremia due to MBL-producing CRE (13). Thus, CRE should be the primary organisms to consider testing for susceptibility to the ATM-CZA combination. In addition, CRE isolates that are susceptible to ATM or CZA alone should not be considered for ATM-CZA combination testing. Depending on the tests available, clinical laboratories can use conventional or direct from specimen phenotypic, genotypic, and/or rapid molecular assays for organism detection (*Enterobacterales* species), susceptibility testing (ATM and CZA resistant), and detection of carbapenemase production to limit the use of combination testing to MBL-positive, CRE isolates (25, 26).

The limited *in vitro* activity of the ATM-CZA combination against MBL-producing P. aeruginosa is likely due to additional noncarbapenemase resistance mechanisms like mutations in porins or efflux pumps (27–30). P. aeruginosa was the most important source of errors in our study, accounting for all ME and a majority of MI from SX and SS (Table 4; Table S1). The VIM-harboring P. aeruginosa isolate (0239), which is susceptible to ATM alone, was the source of all ME for SX and SS (Table S1). While the testing methods displayed utility for GES-producing P. aeruginosa, it should be noted that these two strains are closely related sequence type 309 (ST309) clones and results may not be generalizable to other GES-positive P. aeruginosa isolates. In addition, there are currently no widely available methods for the clinical laboratory to detect the presence of GES enzymes. Thus, our results suggest that the ATM-CZA combination testing methods we evaluated should not be employed routinely for carbapenem-resistant P. aeruginosa isolates.

While most of the synergy-positive isolates included in the study had a clear change from very high ATM MICs to low MICs in the presence of CZA, one isolate did not. The ATM MIC of the E. coli strain 1055 dropped from >64 μg/ml to the intermediate breakpoint MIC (8 μg/ml) in the presence of CZA, while the rest of the synergy-positive strains showed a drop to low susceptible MICs (mode, 0.5 μg/ml) based on the CLSI M100 breakpoints for *Enterobacterales* and P. aeruginosa (31) (Table 1; Table S1). However, based solely on the FIC value of 0.125, the isolate 1055 would be synergy positive. This isolate demonstrates an interesting nuance in our combination testing. While an isolate may have a significant reduction in MIC, it may not meet the threshold for clinical effectiveness. However, defining clinical effectiveness on the basis of an *in vitro* test can be very difficult, especially given the limited treatment options for infection due to CRE and the paucity of clinical studies assessing efficacy of the ATM-CZA combination. The optimal pharmacodynamic/pharmacokinetic (PK/PD) target for the ATM-CZA combination is unknown. Based on a recent prospective observational study and clinical cases, coadministration of a standard dosage of CZA (2.5 g every 8 h) and ATM (2 g every 8 h) was adequate to obtain favorable clinical responses (10, 13). Thus, we used CLSI M100 clinical breakpoints for ATM and CZA, which are based on a >90% probability of PK/PD target attainment with the standard dosage (31). Further studies optimizing the dosing regimen of ATM and CZA may be able to provide information on the clinical benefit of treating an infection due to an isolate with an intermediate ATM MIC of 8 μg/ml in the presence of CZA. This observation demonstrates the potential advantage of methodologies that determine an MIC, which might provide valuable information to guide clinical treatment decisions.

There is a dire need for clinical microbiology diagnostics that emphasize accessibility and affordability in addition to performance and automation since barriers encountered in low-resource settings are often neglected in diagnostic development. The DE method, with 100% sensitivity and specificity, is particularly valuable in low-resource settings that routinely use disk diffusion for susceptibility testing due to affordability and availability of supplies. It is also a low complexity test with an easy visual interpretation of results (growth/no growth) (Fig. 1) and short hands-on time (5 to 10 min). The SX and SS methods, although more expensive, could be the test(s) of choice for a clinical lab that already has established workflows for susceptibility testing using gradient strips. As mentioned earlier, SS and SX can be error prone due to technical aspects of overlaying the gradient strips.

These methods need to be further evaluated in a large multicenter validation study with more isolates harboring diverse β-lactamases and different manufacturer brands of disks, MH broth, and agar to confirm the accuracy, precision, and reproducibility of the DE, SX, and SS methods. It also remains to be determined if these methods are valuable for other organisms, such as nonfermenting Gram-negative bacilli like Stenotrophomonas maltophilia and Acinetobacter spp. Clinical outcome studies are also needed to determine the correlation of these methods with microbiological and clinical outcomes of the ATM-CZA combination. Lastly, the precise concentration of antibiotics eluted from the disk into the MH broth was not confirmed with a biochemical assay, but the bioassay indicated that the QC MIC values were in concordance with those from the mBMD.

In summary, we show that three of the four methods assessed in our study, namely, disk elution, strip crossing, and strip stacking, are each capable of producing accurate results and are practical methods for clinical labs to use for performing ATM-CZA combination testing. These methods may allow the microbiology laboratory to inform clinical decision making for the treatment of severe infections caused by extensively drug-resistant *Enterobacterales.*

## MATERIALS AND METHODS

### Bacterial isolates.

A total of 16 representative isolates were evaluated consisting of 8 *Enterobacterales* and 8 P. aeruginosa strains harboring NDM, VIM, IMP, OXA-48, KPC, or GES (Table 1). Of these isolates, NDM-harboring E. coli (2769) and K. pneumoniae (number 2770) and GES-harboring P. aeruginosa (PA_HTX1) were clinical strains characterized by whole-genome sequencing in previous case studies where a microbiological cure was achieved with the ATM-CZA combination in the setting of bacteremia (7, 10). The remainder of the isolates were obtained from the CDC-FDA Antimicrobial Resistance Isolate Bank (AR Bank) or were patient isolates in our collection that had a defined resistance phenotype and were whole-genome sequenced with the presence of an enzyme confirmed by PCR. E. coli strain ATCC 25922 was included in the study for quality-control purposes. All retrospective isolates were stored at –80°C and subcultured twice on sheep’s blood agar prior to testing.

### Study design.

SX, SS, DE, and DS were the four combination testing methods evaluated against reference mBMD using the combination of ATM with CZA. Testing of all the methods was performed in parallel in a single laboratory using the same 0.5 McFarland inoculum. Testing for each strain was performed in biological triplicate for each method, over three separate days. Single-manufacturer disks (BD, BBL, Sensi-disc), MH broth (Difco, BD), and MH agar (BD, BBL) along with two gradient strip manufacturers (Etest, bioMérieux; MTS, Liofilchem) were used in the study. All three triplicate results were included in the qualitative and quantitative performance analysis. If an isolate yielded a replicate result for the SX, SS, and DE methods that was out of CA with the first replicate, it was repeated. If repeat testing confirmed the initial observations, the result was included in the analysis as a CA error.

The breakpoints used to interpret the MICs were from the CLSI M100 (20) (Table 2). All isolates were categorized as either synergy positive or synergy negative for the performance study based on the mBMD results (Table 1). Synergy positive isolates were those that were ATM and CZA resistant but susceptible to the ATM-CZA combination. Synergy negative isolates were those that were resistant to ATM, CZA, and the ATM-CZA combination or strains susceptible to ATM and/or CZA alone and so by default were susceptible to ATM-CZA. For analysis, MICs between the typical log_2_ dilution MICs determined by Etest or MTS were rounded up to the nearest log_2_ dilution that corresponded with a dilution in the mBMD range.

### Reference modified broth microdilution.

mBMD was used as a reference method for the performance assessment and was performed following CLSI recommendations on in-house-prepared 96-well plates using MH broth (Difco, BD, Sparks, MD). MICs of ATM, CZA, and ATM in the presence of a stable concentration of CZA (at 4 μg/ml of avibactam to match concentration of gradient test strips) were determined from concentrations spanning a doubling dilution range of 0.25 μg/ml to 64 μg/ml. All antimicrobial powders were obtained from Sigma-Aldrich. Quality control (QC) for each run was performed using the E. coli strain ATCC 25922. The CZA and ATM combination was considered effective by mBMD if a strain was resistant to both CZA and ATM alone but categorized as ATM susceptible or intermediate in the presence of a stable concentration of CZA (4 μg/ml avibactam) with an MIC reduction of greater than one doubling dilution.

### Gradient strip cross and strip stacking methods.

Susceptibility testing with Etest (bioMérieux) and MTS (Liofilchem) strips was performed on MH agar (BBL, BD, Sparks, MD) according to the manufacturer’s instructions. For the SX method, plates were inoculated, the ATM strip was placed directly on the agar surface, and the CZA strip was crossed perpendicular to the ATM strip at the intermediate breakpoint (8 μg/ml for *Enterobacterales* and 16 μg/ml for P. aeruginosa) to allow for clear visualization of the susceptible breakpoint (Fig. 1A). For the SS method, plates were inoculated and the ATM strip was placed on the MH agar for 10 min to allow for diffusion of the antimicrobial and removed prior to placing the CZA strip in the same location. The ATM strip was then placed on top of the CZA strip to allow determination of the ATM MIC (Fig. 1A). A strain was considered synergy positive by the SX and SS methods if it was both ATM and CZA resistant and the ATM MIC was interpreted as susceptible or intermediate in the presence of the CZA strip. We set up the individual ATM and CZA gradient strips alongside the combination testing condition to confirm the results of the mBMD, but this was not essential for interpretation of the combination testing.

### Disk elution method.

The DE method was performed with 2 ml of MH broth added to a sterile culture tube, followed by addition of 1 disk of ATM (BD, BBL, Sensi-Disc) and 1 disk of CZA (BD, BBL, Sensi-Disc). The tube was incubated at room temperature for 30 min to allow the antimicrobials to elute from the disk. A 0.5 McFarland standard inoculum was prepared by suspending fresh colonies from an overnight sheep’s blood agar plate in normal saline. Then, 12 μl of this suspension was added to the tube with the eluted disks and vortexed to reach a final inoculum of around 1.5 × 10^5^ CFU per ml. The results were read visually in comparison to a clear broth control after a 16- to 20-hour incubation at 35°C (Fig. 1C). We compared single-disk and double-disk elution in 2 ml of MH broth with representative isolates tested over a 3-day period and found no difference in results. Thus, single-disk testing was used (Table S3). A strain was considered synergy positive if it was ATM and CZA resistant, as determined by mBMD, with no growth observed in the tube with the combination of ATM and CZA disks (Fig. 1C). The tubes with an ATM or a CZA disk alone were observed for informational purposes but were not needed to be set up for interpretation of the results.

### Disk stacking method.

The DS method was performed following CLSI recommendations by placing the CZA disk directly on top of an ATM disk placed on an inoculated MH agar plate with the zone diameter read and interpreted according to ATM breakpoints. A strain was considered synergy positive if it was ATM and CZA resistant, as determined by mBMD, with the ATM zone diameter interpreted as susceptible or intermediate in the presence of the CZA disk on top (Fig. 1B). We placed an ATM disk alone and CZA disk alone on the same plate for informational purposes, although only the ATM-CZA disk stack zone diameter was read to determine if the ATM-CZA combination was synergistic.

### Reproducibility study.

A reproducibility analysis was performed to evaluate variation within the same laboratory site (CARMiG, UTHealth) on all the triplicate results for each isolate, which was performed on three separate testing days. Essential agreement was calculated by determining if each replicate result of a strain per method was within one doubling dilution of the mode of the three replicate results (Table 5). If there was no obvious mode with 3 different numerical results, the median was used instead.

### Fractional inhibitory concentration (FIC) score calculation.

The FIC score was determined using MIC values generated by mBMD, SX, and SS. The FIC scores indicated in Table 3 for each strain are an average of the calculated score of each triplicate. When the ATM-CZA combination yielded a FIC score of <0.5, then it was considered synergistic, while a score ≥0.5 indicated that the combination was additive or indifferent relative to ATM or CZA alone.

### Qualitative performance analysis.

The result of each test was interpreted as synergy positive or synergy negative, as defined above in the study design section, and compared with the reference mBMD method. For example, a result was considered a “true positive” if both the test and mBMD called an isolate synergy positive. The precise MIC value or zone diameter reading for SX, SS, and DS did not have any bearing on this analysis. The sensitivity, specificity, and 95% confidence interval were calculated for each test.

### Quantitative error-rate bound analysis.

This assessment was performed only on SX, SS, and DS methods. The categorical agreement (CA) and essential agreement (EA; MIC ± one doubling dilution) were evaluated with very major errors (VMEs), major errors (MEs), and minor errors (MIs) calculated (20). The evaluable EA included only isolates with a defined MIC value and discarded isolates below or above the lowest and highest drug concentration on mBMD panels. CA was defined using the CLSI breakpoints as the agreement of interpretative results between the method under evaluation and mBMD. Discrepancies between the method under evaluation and mBMD were categorized as follows: VME, false-susceptible result under the test method and resistant by mBMD; ME, false-resistant resistant by test method and susceptible by mBMD; and MI, a discrepancy between the test and reference methods involving an intermediate or nonsusceptible result.
