# Supplementary material for: Evaluation of Susceptibility Testing Methods for Aztreonam and Ceftazidime-Avibactam Combination Therapy on Extensively Drug-Resistant Gram-Negative Organisms
Source: Antimicrob Agents Chemother. 2021 Oct 18;65(11):e00846-21. doi: 10.1128/AAC.00846-21 (PMC8522751; doi:10.1128/AAC.00846-21)
Supplement: Supplemental file 1 — Supplemental tables and figures. Download AAC.00846-21-s0001.pdf, PDF file, 0.4 MB [file aac.00846-21-s0001.pdf]

Supplementary Table 1. Summary of reproducibility of the Disk elution, Strip stacking and Strip cross methods to determine efficacy of the CZA and ATM combination against *Enterobacterales* and *P. aeruginosa.* Data listed as readings of the results from broth microdilution (BMD), disk elution (DE), Strip Stacking and Strip Crossing methods on day 1,2, and 3. DE reading indicated as growth (+) or no growth (-). Mode (M) of the biological replicates indicated. When no clear mode exists, a mode is assigned by rounding the closest reading up by one doubling dilution to match another reading. Aztreonam (A), ceftazidime avibactam (C), aztreonam + ceftazidime avibactam (A +C) at 4 mcg/ml of avibactam.

		**BMD MIC (mcg/ml)**				**DE**			**Strip Stacking**								**Strip Crossing**							
									**Etest**				**MTS**				**Etest- A + C**				**MTS- A + C**			
		**1**	**2**	**3**	**M**	**1**	**2**	**3**	**1**	**2**	**3**	**M**	**1**	**2**	**3**	**M**	**1**	**2**	**3**	**M**	**1**	**2**	**3**	**M**
**ATCC**	A	≤0.25	≤0.25	≤0.25	≤0.25	-	-	-	0.125	0.125	0.125	0.125	0.064	0.064	0.094	0.064	0.125	0.125	0.125	0.125	0.094	0.094	0.064	0.094
	C	≤0.25	≤0.25	≤0.25	≤0.25	-	-	-	0.094	0.125	0.125	0.125	0.094	0.094	0.094	0.094								
	A+C	≤0.25	≤0.25	≤0.25	≤0.25	-	-	-	0.023	0.06	0.06	0.06	0.023	0.047	0.047	0.047								
EC #2769	A	>64	>64	>64	>64	+	+	+	>256	>256	>256	>256	>256	>256	>256	>256	3	4	2	3	3	2	3	3
	C	>64	>64	>64	>64	+	+	+	>256	>256	>256	>256	>256	>256	>256	>256								
	A+C	4	2	2	2	-	-	-	1	1.5	0.094	1	0.094	0.023	1	0.5								
KP #2770	A	>64	>64	>64	>64	+	+	+	>256	>256	>256	>256	64	64	128	64	0.5	0.75	1.5	1	1	1	0.5	1
	C	>64	>64	>64	>64	+	+	+	>256	>256	>256	>256	>256	>256	>256	>256								
	A+C	2	0.5	0.5	0.5	-	-	-	0.19	0.125	0.125	0.125	0.094	0.064	0.125	0.094								
PA HTX_1	A	>64	>64	>64	>64	+	+	+	>256	>256	>256	>256	>256	>256	>256	>256	4	6	8	6	8	6	8	8
	C	>64	64	32	48	+	+	+	>256	48	96	>256	>256	>256	>256	>256								
	A+C	4	8	4	4	-	-	-	8	8	6	8	4	8	8	8								
PA HTX_70	A	16	16	16	16	+	+	+	24	24	32	24	16	16	16	16	16	16	16	16	8	16	8	8
	C	64	64	64	64	+	+	+	96	64	96	96	>256	>256	>256	>256								
	A+C	16	16	16	16	+	+	+	16	12	24	17	16	12	16	16								
PA HTX_133	A	>64	64	64	64	+	+	+	>256	>256	>256	>256	>256	>256	>256	>256	8	8	8	8	8	8	6	8
	C	32	32	16	32	+	+	+	>256	>256	>256	>256	>256	>256	>256	>256								
	A+C	4	8	4	4	-	-	-	8	8	8	8	6	6	4	6								
KP #622	A	≤0.25	≤0.25	≤0.25	≤0.25	-	-	-	0.016	0.023	0.016	0.016	0.016	0.023	0.016	0.016	0.016	0.016	0.016	0.016	0.016	0.016	0.016	0.016
	C	32	32	16	32	+	+	+	4	6	6	6	6	8	6	6								
	A+C	≤0.25	≤0.25	≤0.25	≤0.25	-	-	-	0.016	0.016	0.016	0.016	0.016	0.016	0.016	0.016								
EClo #1042	A	>64	>64	>64	>64	+	+	+	>256	>256	>256	>256	>256	>256	>256	>256	0.5	1.5	2	1.5	1.5	1.5	0.75	1.5
	C	>64	>64	>64	>64	+	+	+	>256	>256	>256	>256	>256	>256	>256	>256								
	A+C	1	1	0.5	1	-	-	-	0.75	1	1	1	0.38	0.75	0.75	0.75								
	A	8	8	8	8	+	-	-	12	12	12	12	8	6	12	8	8	12	8	8	8	8	8	8

***PA* #0241**	C	>64	>64	>64	>64	+	+	+	>256	>256	>256	>256	>256	>256	>256	>256								
	A+C	8	8	8	8	-	-	-	8	12	8	8	12	12	12	12								
***EC* #1055**	A	>64	>64	>64	>64	+	+	+	>256	>256	>256	>256	>256	>256	>256	>256								
	C	>64	>64	>64	>64	+	+	+	>256	>256	>256	>256	>256	>256	>256	>256	16	8	4	10	6	6	8	6
	A+C	8	8	8	8	-	-	-	12	16	24	16	6	8	6	6								
***EC* #1057**	A	>64	>64	>64	>64	+	+	+	>256	>256	>256	>256	16	12	16	16								
	C	>64	>64	>64	>64	+	+	+	>256	>256	>256	>256	>256	>256	>256	>256	4	3	4	4	2	2	2	2
	A+C	4	4	2	4	-	-	-	1.5	3	3	3	1.5	1.5	2	1.5								
***KP* #1063**	A	>64	>64	>64	>64	+	+	+	>256	>256	>256	>256	>256	>256	>256	>256								
	C	>64	>64	>64	>64	+	+	+	>256	>256	>256	>256	>256	>256	>256	>256	1.5	1.5	1	1.5	2	1.5	1.5	1.5
	A+C	1	0.5	0.5	0.5	-	-	-	1	0.75	1	1	0.5	1	1	1								
***PA* #0246**	A	>64	>64	>64	>64	+	+	+	>256	>256	>256	>256	>256	>256	>256	>256								
	C	>64	>64	>64	>64	+	+	+	>256	>256	>256	>256	>256	>256	>256	>256	>256	>256	>256	>256	>256	>256	>256	>256
	A+C	>64	64	64	64	+	+	+	>256	>256	>256	>256	>256	>256	>256	>256								
***PA* #0250**	A	>64	>64	>64	>64	+	+	+	>256	>256	>256	>256	>256	>256	>256	>256								
	C	>64	>64	>64	>64	+	+	+	>256	>256	>256	>256	>256	>256	>256	>256	>64	>64	>64	>64	>256	>256	>256	>256
	A+C	>64	>64	>64	>64	+	+	+	>256	>256	>256	>256	>256	>256	>256	>256								
***PA* #0239**	A	8	8	8	8	+	+	+	48	32	16	32	16	16	16	16								
	C	>64	64	64	64	+	+	+	>256	>256	>256	>256	>256	>256	>256	>256	32	4	24	20	8	8	8	8
	A+C	4	8	4	4	-	-	-	32	16	6	18	16	12	16	16								
***PA* #0249**	A	16	16	16	16	+	+	+	12	24	24	24	8	24	24	24								
	C	>64	>64	>64	>64	+	+	+	>256	>256	>256	>256	>256	>256	>256	>256	8	16	12	12	8	12	12	12
	A+C	16	8	16	16	+	+	+	6	24	12	12	8	16	32	20								
***KP* #1041**	A	64	>64	64	64	+	+	+	24	32	32	32	16	24	24	24								
	C	2	2	2	2	-	-	-	1	2	1	1	0.75	0.75	0.75	0.75	1	1	1	1	1	1	1	1
	A+C	1	1	1	1	-	-	-	0.38	0.5	0.5	0.5	0.25	0.25	0.25	0.25								

Supplementary Table 2. Comparison of disk elution results for *P. aeruginosa* when using 1 ATM and CZA disk versus 2 ATM and CZA disks each to , eluted in 2 ml of Mueller Hinton broth. 1 ATM and/or CZA disk in 2 ml of broth yields a final active concentration of approximately 15 ug of the antimicrobial while 2 ATM and/or CZA disks yield a final concentration of approximately 30 ug of the antimicrobial. Note: The resistant breakpoint as per CLSI M100 guidelines for *P. aeruginosa* is 32 mcg/ml.

**Strain**	**1 ATM + CZA disk**			**2 ATM + CZA disks**		
	Day 1	Day 2	Day 3	Day 1	Day 2	Day 3
**ATCC**	-	-	-	-	-	-
EC #2769	-	-	-	-	-	-
KP #2770	-	-	-	-	-	-
E. clo #1042	-	-	-	-	-	-
PA HTX_1	-	-	-	-	-	-
PA #0239	-	-	-	-	-	-
PA HTX_133	-	-	-	-	-	-
PA #0241	-	-	-	-	-	-
PA #0246	+	+	+	+	+	+
PA #0250	+	+	+	+	+	+

Supplemental figures

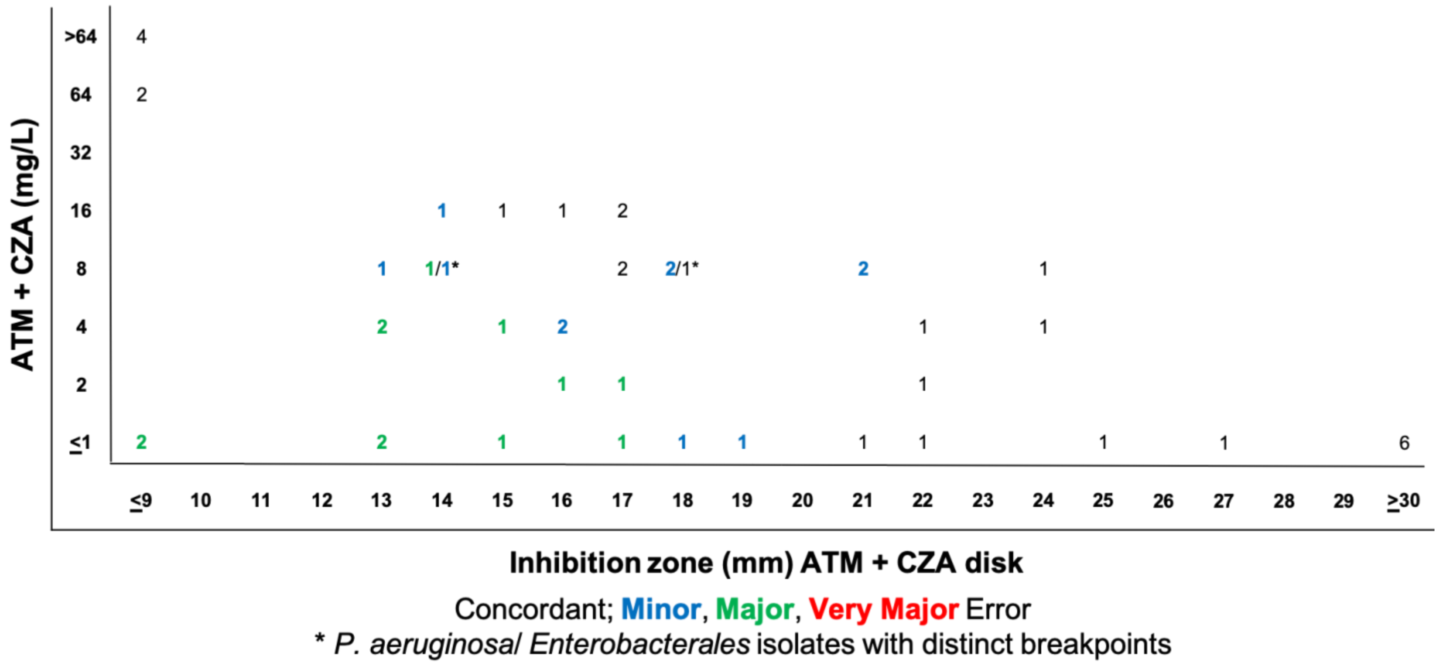

Figure S1. Modified broth microdilution (mBMD) and disk stacking distributions of zone diameters for ATM disk in presence of CZA disk on top, measured to assess synergy. Errors based on categorical disagreement are indicated by colors. * # of *P. aeruginosa* isolates/ # of *Enterobacterales* isolates listed in that order, separated by a back slash; this is to distinguish isolates with errors since ATM breakpoints for are different for *Pseudomonas* and *Enterobacterales* spp.

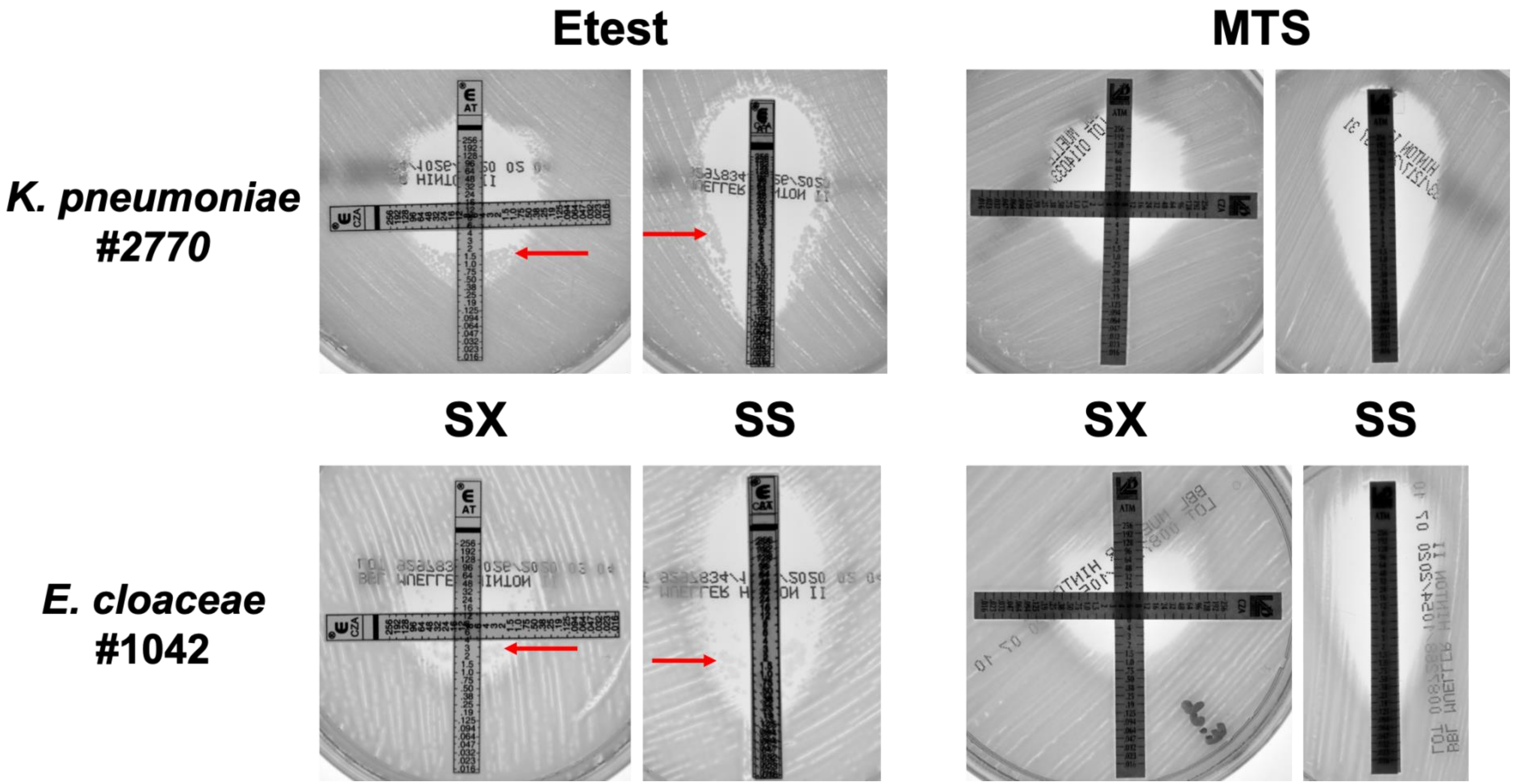

Figure S2. SX and SS methods with two brands of gradient strips- MTS from Liofilchem and Etest from bioMerieux demonstrating variation in inner zone colony morphology for two representative *Enterobacterales* isolates. Red arrow indicates colonies that are distinct from the inocula lawn within the zone of clearance for Etest while a homogenous phenotype was observed for MTS.
